# Brood size moderates associations between relative size, telomere length, and immune development in European starling nestlings

**DOI:** 10.1002/ece3.2551

**Published:** 2016-10-17

**Authors:** Daniel Nettle, Clare Andrews, Sophie Reichert, Tom Bedford, Annie Gott, Craig Parker, Claire Kolenda, Carmen Martin‐Ruiz, Pat Monaghan, Melissa Bateson

**Affiliations:** ^1^ Institute of Neuroscience and Centre for Behaviour & EvolutionNewcastle UniversityHenry Wellcome BuildingNewcastleUK; ^2^ Institute of Biodiversity, Animal Health and Comparative MedicineUniversity of GlasgowGlasgowUK

**Keywords:** body size, ecological immunology, growth, Sturnus vulgaris, starlings, telomeres

## Abstract

For young birds in a nest, body size may have implications for other aspects of development such as telomere length and immune function. However, it is possible to predict associations in either direction. On the one hand, there may be trade‐offs between growth and telomere maintenance, and growth and investment in immune function, suggesting there will be negative correlations. On the other hand, relatively larger individuals might be advantaged in competition with their nest‐mates, allowing them to garner more resources overall, leading to positive correlations. We studied development over the nestling period in 34 nests of wild European starlings, *Sturnus vulgaris*. Intrabrood competition is typically more intense in larger broods. Hence, we predicted that body size should become an increasingly positive predictor of telomere length and immune functioning as brood size increases. In partial support of our prediction, there were significant interactions between brood size and body size in predicting both erythrocyte telomere length change and plasma levels of the cytokine interleukin‐6. The associations between body size and these outcomes went from negative in the smallest broods to positive in the largest. A further immune marker, high‐sensitivity C‐reactive protein, showed no systematic patterning with body size or brood size. Our results confirm that the size to which a nestling grows is important for telomere dynamics and the development of the immune system, but the phenotypic associations are moderated by the competitive context.

## 1 Introduction

In altricial birds, there is mounting evidence that the size to which a nestling grows has implications for both the length of telomeres and the development of immune function. An intriguing feature of this topic is that, a priori, it is possible to plausibly predict associations in either direction: Larger body size at the end of the nestling period might be associated with shorter telomeres and poorer immune function; or larger body size might be associated with longer telomeres and better immune function.

Telomeres are repetitive noncoding DNA sequences found at the ends of eukaryotic chromosomes that play an important role in genome stability (Hoeijmakers, 2001). They shorten largely as a consequence of loss during cell division. Some of this loss is an inevitable product of the incomplete replication of the lagging strand during DNA replication, but the amount of loss can be substantially increased by oxidative damage (Von Zglinicki, 2002). Growing to a larger size requires more cell division. Individuals growing to a larger size typically do so by growing faster, and a faster rate of growth may also increase oxidative damage. To repair damage to telomeres is assumed to carry some cost. Thus, other things being equal, there should be a trade‐off between body size at the end of the fledgling period and telomere length (Reichert et al., 2014). In accordance with this possibility, several recent avian studies have observed associations between larger body size or faster growth and shorter telomere length at the end of development (Herborn et al., 2014; Noguera, Metcalfe, Boner, & Monaghan, 2015; Ringsby et al., 2015). In addition, Ringsby et al. (2015) showed, in house sparrows, that selection for increased body size led to a reduction in mean telomere length. The existence of a negative body size–telomere length relationship is consistent with the pattern often observed within populations that larger individuals tend to live less long (Bartke, 2012; Bernstein, 2010; Miller, Harper, Galecki, & Burke, 2002), given that shorter telomeres at the end of development are associated with reduced longevity (Boonekamp, Mulder, Salomons, Dijkstra, & Verhulst, 2014; Heidinger et al., 2012).

Related logic can be applied to immune functioning. The vertebrate immune system is a complex set of defense mechanisms against pathogens and irritants. It is costly to develop and deploy (Hasselquist & Nilsson, 2012; Sheldon & Verhulst, 1996). In altricial birds, immune activity develops rapidly over the course of the nestling period, but does not reach adult levels until after fledging (Killpack & Karasov, 2012; Stambaugh, Houdek, Lombardo, Thorpe, & Caldwell Hahn, 2011). Immune expression during the nestling period, particularly the expression of induced immune responses, is resource limited (Birkhead, Fletcher, & Pellatt, 1999; Killpack, Elijah, & Karasov, 2015). That is, differences between nestlings in the magnitude of immune markers primary reflect the energetic investment that has been made in the development of immune function. Thus, for individuals with a fixed level of resources available, there is a trade‐off: Investing more in growth reduces energy available for investment in immune development. There is compelling evidence from selection experiments for an evolutionary trade‐off between growth rate and immune function (Van Der Most, De Jong, Parmentier, & Verhulst, 2011). Moreover, in blue tit nestlings, when immune competence is experimentally upregulated through methionine supplementation, body size is reduced (Brommer, 2004). Thus, we might predict that the body size to which nestlings grow would be negatively correlated with their immune development.

These predictions of negative phenotypic correlations rely on the assumption that there is no systematic inequality in available resources across nestlings of different sizes. However, this is not necessarily the case. Within a nest, larger nestlings are favoured by parents in provisioning and have to beg less to receive food (Cotton, Wright, & Kacelnik, 1999; Kilner, 1995; Mock, Schwagmeyer, & Dugas, 2009). Begging is itself costly for growth (Kilner, 2001). Thus, nestlings that are already larger than their competitors will enjoy more resource input for lower cost, allowing them to invest more in nongrowth functions such as antioxidant defenses and immune functioning, while nestlings that are already smaller are caught in a feedback loop of escalating begging costs just to obtain the energy to avoid falling even further behind. From this perspective, we might expect that larger nestlings will be *better* off in terms of telomere length and immune capacity, leading to positive rather than negative associations. There is evidence for such positive associations. In a correlational study of a small passerine, the thorn‐tailed rayadito, there was a positive relationship between body mass and telomere length toward the end of the nestling period, mediated by increased corticosterone in lighter nestlings (Quirici, Guerrero, Krause, Wingfield, & Vásquez, 2016). Boonekamp et al. (2014) experimentally reduced or enlarged jackdaw broods. They found that greater fledging mass was associated with longer telomeres at day 30 once initial telomere length was controlled for, although this was true only in the enlarged broods. In a previous study on European starlings, we composed small (two nestling) or large (seven nestling) broods experimentally. In the large broods, we found a correlation between position in the within‐brood size hierarchy and the rate of telomere change: The relatively larger chicks showed less telomere loss during development (Nettle, Monaghan, Boner, Gillespie, & Bateson, 2013). We were able to confirm that it is position in the size hierarchy of the brood (i.e., relative rather than absolute size) that matters in a subsequent experiment (Nettle et al., 2015) where we manipulated this directly (see also Stier, Massemin, Zahn, Tissier, & Criscuolo, 2015). For immune capacity, likewise, correlational studies across a number of species have found that larger size goes with higher, rather than lower, immune capacity (Fair, Hansen, & Ricklefs, 1999; Hoi‐Leitner, Romero‐Pujante, Hoi, & Pavlova, 2001; Lochmiller, Vestey, & Boren, 1993; Saino, Calza, & Moller, 1997; Snoeijs, Pinxten, & Eens, 2005; Tella, Bortolotti, Forero, & Dawson, 2000).

Whether negative or positive correlations between body size and telomere length/immune development will be seen may depend on the harshness of the conditions under which growth takes place. In particular, the intensity of intrabrood competition should be an important determinant. A simple marker of the intensity of intrabrood competition is brood size itself. As brood size increases, the per nestling food supply is reduced (Rhymer, Devereux, Denny, & Whittingham, 2012; Saino et al., 1997) and begging increases (Wright & Cuthill, 1990a). Intrabrood inequality in weight gain also tends to become greater, as competition and parental favoritism create winners and losers (Nettle et al., 2013; Wright & Cuthill, 1990b). Thus, in large broods, size‐based competitive advantage tends to become important; within such broods, we should expect positive associations between body size and telomere length and body size and immune development, with the larger chicks in the brood having longer telomeres than their disadvantaged siblings. In small broods on the other hand, size‐based competitive advantage tends to be less important; here the negative associations—larger individuals having shorter telomeres—are more likely to be detectable. The possibility that brood size moderates the consequences of body size is already suggested within previous experimental studies by the fact that larger body size was associated with less telomere loss only in enlarged broods (Boonekamp et al., 2014; Nettle et al., 2013). However, the possibility has not yet been tested systematically across the range of natural brood sizes nor extended from telomere dynamics to immune parameters.

In this study, we measured nestling body size, telomere length change, and two markers of immune function in European starling nestlings raised in unmanipulated broods of naturally varying sizes. We measured erythrocyte telomere length as early in life as feasible (3 days after hatching), and again 15 days after hatching, at which point, the birds were close to adult weight and around 5 days from fledging. Based on our previous studies, we expected that there would be substantial continuity of telomere length across the time points, as there is consistent interindividual variation in starting telomere length, but that we would nonetheless be able to detect significant within‐individual shortening between the two time points. Our two immune markers were interleukin‐6 (IL‐6) and C‐reactive protein, which we measured with a high‐sensitivity assay (hsCRP). IL‐6 is a pro‐inflammatory cytokine involved early in the mobilization of the innate immune response (Zimmerman, Bowden, & Vogel, 2014). It has been previously used in passerine birds as a marker of response to experimental antigen challenge in adults (Adelman, Bentley, Wingfield, Martin, & Hau, 2010). C‐reactive protein is produced in plasma during the acute‐phase immune response. Its synthesis is immediately regulated by IL‐6 (Pepys & Hirschfield, 2003). In birds, its previous use is largely restricted to studies of response to infection in the chicken (e.g., Abd El‐Hamid, Ahmed, Sadek, & Ellakany, 2014). Note that we are interpreting these markers in a different way from their usual use in epidemiological studies of adult humans, where high unstimulated levels would indicate chronic inflammation and suggest poorer health status (e.g., Nettle, 2014). In the present context, we are instead taking a higher level to indicate more advanced development of immune capacity, in accordance with previous findings on immune development in nestling birds.

As described above, our main hypothesis was that brood size would moderate the associations between body size on the one hand and telomere change and immune function on the other, with positive associations within the larger broods where intrabrood competition is intense, and negative associations within the smaller broods where intrabrood competition is relaxed. Thus, there should be significant interactions between brood size and body size in predicting both developmental telomere change and the immune measures. The basis for our predictions would be undermined if brood size was not a reliable marker of the intensity of intrabrood competition, for example, because parents that laid larger clutches were of higher provisioning ability than those that laid small clutches. Thus, it was important to test whether average resources per nestling decreased with increase in brood size.

## 2 Methods

### 2.1 Study system and procedure

We studied all the nests we found at previously installed nest box colonies on four farms in Northumberland, England, during April and May 2015. Nest boxes were checked regularly to establish dates of laying and hatch. On day 4 from first hatching (where day 1 indicates day of first hatching), all nestlings were briefly removed from the nest box and weighed using a digital balance to a precision of 0.1 g, and a blood sample of approximately 70 μl was taken from the medial metatarsal vein using a 25‐gauge needle and plain glass capillary tube. Nestlings were individually marked using colored electrical tape and returned to the nest. The nests were revisited on day 7 for weighing and to replace the electrical tape with colored plastic leg rings. On day 16, the nest was visited again. A further weight was taken, and the right tarsus measured using digital callipers to a precision of 0.01 mm. (Tarsi were not measured at day 4 or day 7 to minimize time out of the nest for small nestlings.) On day 16, a second blood sample (approximately 120 μl, from either alar or medial metatarsal vein) was also taken, and the plastic leg ring replaced with a permanent numbered metal ring. All blood samples were placed in prelabeled EDTA‐treated sample tubes (Sarstedt AG & Co, Numbrecht, Germany, catalog no. 20.1278) and immediately put on ice. Within 6 hr, erythrocytes were separated from plasma by centrifuging and both components frozen at −80°C for later analysis.

### 2.2 Telomere measurement

DNA was extracted from erythrocytes using a KingFisher™ Flex Magnetic Particle Processor (Thermo Scientific). We verified quality and purity of extracted DNA with a NanoDrop (Thermo Scientific) spectrophotometer (absorbance ratio A260/280 > 1.7; A260/230 > 1.8). Relative telomere length was assessed by quantitative real‐time PCR amplification (qPCR; Cawthon, 2002) and expressed as T/S ratio, that is, the ratio of starting template amounts of telomeric sequence and GAPDH as reference single‐copy gene. T/S ratios were calculated using the ΔΔCt method. Calculation using the approach of Pfaffl (Pfaffl, 2001), which incorporates variation in amplification efficiency, produced virtually identical results (*r* > .99). The T/S ratio is widely used as a measure of relative telomere length and is suitable for summarizing within‐individual changes in telomere length (Nussey et al., 2014); we henceforth refer to it as simply telomere length.

Forward and reverse primers for the GAPDH gene were 5′‐AAACCAGCCAAGTACGATGACAT ‐3′ and 5′‐CCATCAGCAGCAGCCTTCA‐3′, respectively. These primers were developed in the zebra finch (Criscuolo et al., 2009) but have been used in other passerine species including the starling (Nettle et al., 2013, 2015). Telomere primers were as follows: Tel1b (5′‐CGGTTTGTTTGGGTTTGGGTTTGGGTTTGGGTTTGGGTT‐3′) and Tel2b (5′‐GGCTTGCCTTACCCTTACCCTTACCCTTACCCTTACCCT‐3′). qPCRs for both telomere sequences and GAPDH were performed using 10 ng of DNA with both sets of primers, in a final volume of 25 μl containing 12.5 μl of Absolute blue qPCR SYBR green Low Rox master mix (Thermo scientific). Primer concentrations in the final mix were 500 nmol/L for the telomere assay and 70 nmol/L for GAPDH. Real‐time amplification of telomere sequences and GAPDH was performed on separate 96‐well plates. The telomere thermal profile was 15 min at 95°C, followed by 27 cycles of 15 s at 95°C, 30 s at 58°C, 30 s at 72°C. The GAPDH thermal profile was 15 min at 95°C, followed by 40 cycles of 15 s at 95°C, 30 s at 60°C, 30 s, 72°C. Both assays were followed by melt curve analysis of (58–95°C 1°c/5 s ramp).

Each sample was assayed in triplicate and the mean of the three assays used. Samples were randomly assigned over 14 plates, with both samples (day 4 and day 16) from the same individual on the same plate. Each plate included serial dilutions (telomere and GAPDH—40, 20, 10, 5, 2.5, 1.25 ng) of a pool of DNA from different birds which were run in triplicate. These serial dilutions were used to generate a reference curve on each plate, in order to control for the amplifying efficiency of the qPCR. Mean amplification efficiencies calculated from the reference curves of the qPCR runs were 97%–107% (telomere) and 97%–109% (GADPH). *R*² values calculated from the reference curves of the qPCR runs were 0.98–0.99 for both the telomere and GADPH assays. Both a negative control (water) and a melting curve were run for each plate to check for specific amplification of a unique amplicon and for the absence of primer–dimer artefacts. Intraplate mean coefficients of variation for Ct values were 1.8% (telomere) and 0.3% (GAPDH). Interplate coefficients of variation based on repeated samples were 2.7% (telomere) and 0.7% for (GAPDH). The mean coefficient of variation for the T/S ratios was 17%, which is in line with previously published results (e.g., Bize, Criscuolo, Metcalfe, Nasir, & Monaghan, 2009). There were 40 individuals for which the GAPDH assay failed and which were removed from the analysis. A possible explanation for this is that these individuals carry a mutation in the GAPDH gene which inhibits its amplification. We have encountered the same phenomenon in previous studies of this starling population (Nettle et al., 2013, 2015).

### 2.3 Immune markers

Plasma samples recovered from EDTA blood were assessed for IL‐6 and high‐sensitivity CRP by sequential solid phase sandwich enzyme‐linked immunosorbent assay (ELISA) protocols. Both assays are validated for use on plasma derived from EDTA blood. For IL‐6, samples were brought up to a final volume of 100 μl and applied onto a chicken IL‐6 ELISA Kit (MyBiosource, San Diego, CA, USA). The average plasma dilution factor was 1.79. As the measurements were performed on diluted plasma, the manufacturer's method was adapted for optimal detection of low levels of IL‐6 and the standard curve shifted to lower values (6‐point standard curve from 125 to 3.9 pg/ml). The fit of the standard curve was maintained. Under these changes, one sample displayed IL‐6 levels above the standard curve. For some nestlings, the volume of plasma recovered was very low, and therefore, the plasma dilution to reach the desired volume of sample for the assay (up to a dilution factor of 3.6) proved to be too high to confidently detect IL‐6 levels (lower limit of detection, 1.5647 pg/ml). Briefly, plate wells were loaded with 100 μl of either diluted starling plasma samples standards or blank (sample diluent) and incubated for 90 min at 37°C. The content of the wells was put aside, and the wells with the antibody–antigen complexes were treated with 100 μl of biotinylated detection antibody for 1 hr at 37°C and washed three times prior to incubation with 100 μl of horseradish peroxidase (HRP) conjugate for 30 min at 37°C. After three further washes, the wells were treated with 90 μl of substrate for 15 min at 37°C protecting from light, followed by 50 μl of stop solution to stop the colorimetric reaction. Optical density measurements were taken at 450 nm wavelength on a FLUOstar Omega microplate reader (BMG Labtech, Aylesbury, UK) applying a blank correction followed by a four‐parameter logistic (4‐PL) curve fit to the standard curve. Standards were run in duplicate, and samples, due to the limited plasma volume, were run as a single measurement. An internal control of known IL‐6 concentration (15.6 pg/ml) was included on each plate to correct for plate‐to‐plate variation. The interassay coefficient of variation based on the internal controls was 8.6%. Final values of IL‐6 concentration, after applying the corresponding dilution factor for each sample, were expressed in pg/ml.

For hsCRP, the above diluted starling plasma samples were then applied onto a pigeon hsCRP ELISA Kit (MyBiosource, San Diego, CA, USA). Briefly, plate wells were loaded with 50 μl of either samples, standards (6‐point standard curve from 8 μg/ml to 0.25 μg/ml) or blank (sample diluent) followed by 100 μl of HRP‐conjugated reagent to each well and incubated for 60 min at 37°C. The wells were washed three times and treated with chromogen solutions A and B (50 μl each per well) for 15 min at 37°C. The reaction was stopped with 50 μl stop solution, and optical density measurements were obtained at 450 nm wavelength on a FLUOstar Omega microplate reader (BMG Labtech, Aylesbury, UK) applying a blank correction followed by a four‐parameter logistic (4‐PL) curve fit to the standard curve. Standards were run in duplicate, and samples were run as a single measurement. An internal control of known hsCRP concentration (1 μg/ml) was included on each plate to correct for plate‐to‐plate variation. The interassay coefficient of variation based on the internal controls was 3.1%. Final values of hsCRP concentration, after applying the corresponding dilution factor for each sample, were expressed in μg/ml.

### 2.4 Molecular sexing

Molecular sexing was carried out on the day 16 blood sample for each nestling by amplification of the chromodomain‐helicase‐DNA binding (CHD) genes in 20 μl real‐time qPCR reactions. Final concentrations of reagents were 1X Absolute blue qPCR SYBR green Low Rox master mix (Thermo scientific), 0.8 μM 2550F (5′‐GTTACTGATTCGTCTACGAGA‐3′) (Fridsolfsson & Ellegren, 1999), 0.8 μM 2757R (5′‐AATTCCCCTTTTATTGATCCATC‐3′) (Griffiths, unpublished data), and 10 ng of DNA. The thermal cycle profile for the PCR comprised 95°C for 15 min, followed by 35 cycles 95°C for 45 s, 52°C for 1 min, and 72°C for 1 min. After the qPCR was completed, a melting curve was recorded by holding at 95°C for 1 min, cooling to 45°C for 1 min, heating slowly at 1°C/5s to 95°C, and holding at 95°C for 30 s. Sex was determined by examining the dissociation curve, with two peaks indicating the presence of a Z and W chromosome (female) and one peak indicating the presence of only Z chromosomes (male). Two samples from a known male and a known female were used as controls.

### 2.5 Statistical analysis

Our measure of body size was day 16 weight. The alternative possibility was day 16 tarsus length. This was positively correlated with day 16 weight (*r*_153_ = .46, *p *<* *.01), and results are qualitatively similar if tarsus length is used instead. We also calculated average daily growth rate, that is, the difference between day 16 weight and day 4 weight, divided by 12, but this was strongly correlated with day 16 weight (*r*_153_ = .72, *p *<* *.01), produced the same pattern of results, and hence is not considered further. Our previous findings suggest that what is important in terms of the costs of competition is size relative to other nestlings in the same brood (Nettle et al., 2015). We thus calculated relative weight measures consisting of *z*‐scores for each bird within its nest (i.e., [nestling weight − mean weight of nestlings in the brood] / *SD* of weights in the brood). Relative weight is thus 0 for a nestling that is the average weight for its brood, positive for a relatively large nestling, and negative for a relatively small one, and the variance of relative weight is homogenous across different nests. Relative and absolute weights on day 16 were significantly positively correlated (*r*_153_ = .58, *p *<* *.01). Relative weight on day 16 was also substantially correlated with relative weight on day 7 (*r*_153_ = .55, *p *<* *.01), indicating that nestlings that were relatively small on day 16 tended to have been so for much of the earlier period. To verify that relative weight rather than absolute weight was the better predictor of telomere and immune outcomes, we ran the statistical models using each in turn, and compared model fits, as reported in Results.

To calculate a single measure of telomere length change between day 4 and day 16, we used the D statistic (see Verhulst, Aviv, Benetos, Berenson, & Kark, 2013), which corrects for the regression to the mean that would be expected in successive imperfectly correlated measurements. We calculated D such that a more negative number indicates greater telomere loss. (Note, however, that D = 0 does not represent no change, but rather, corresponds to the average amount of shortening observed in the whole sample.) The distribution of D had a number of outliers in both directions, and thus, we used a signed square root transformation in order to improve residual normality. The D measure thus transformed was highly correlated with telomere length on day 16 (*r*_113_ = .71, *p *<* *.01), and also with the simple difference between telomere length on day 16 and telomere length on day 4 (*r*_113_ = .77, *p *<* *.01). All reported results are similar using the simple difference instead of the D statistic as the outcome measure.

IL‐6 and hsCRP both had right‐skewed distributions. For IL‐6, we used a reciprocal transformation, multiplying by −1 to maintain the same direction of the measure, that is, higher indicates more IL‐6. For hsCRP, we employed a square root transformation.

All analyses used linear mixed models in the “lme4” package (Bates, Machler, Bolker, & Walker, 2015) of the R language (R Core Development Team 2015), with maximum‐likelihood estimation. All models contained random effects of nest. Models with telomere‐related variables as the outcome also contained an additional random effect of plate. The fixed effects structure of each model is specified as they are reported and shown in Table 1. Significance tests for fixed effects were based on the likelihood ratio test (LRT). The criterion for statistical significance was set at *p* = .05. We report marginal *r* ^2^ (Nakagawa & Schielzeth, 2013), an estimate of the variance explained by the fixed predictors in mixed models, for each of the models. Model residuals were checked for heteroscedasticity and transformations of dependent variables adjusted until this was satisfactory. We have not formally accounted for nestling mortality between day 4 and day 16—which may be nonrandomly related to body weight and brood size—in our models. We justify this by the fact that mortality was rare and not completely restricted to the largest broods (see 3.1 Description of sample, in Results). Moreover, selective disappearance was likely to act conservatively in terms of the hypothesis under test. If, for example, the smallest nestlings were the most likely to disappear, this would truncate the observed body size range, militating against detecting any associations between body size and the outcome variables.

**Table 1:** Output from the linear mixed models. Random effects included were nest (all models) and telomere plate (model 2). Relative weight refers to day 16. Parameter estimates (B) and their standard errors (*SE*) are shown

Model	Outcome variable	Fixed predictors	Likelihood ratio test	*p*‐value	B	*SE* (B)
1	Weight day 16	Male sex	13.94	<.01	2.93	0.76
		Brood size	8.78	<.01	−2.58	0.83
2	Telomere length change	Telomere length day 4	0.11	.74	−0.03	0.28
		Male sex	0.14	.71	0.03	0.07
		Brood size	0.01	.93	0.004	0.06
		Relative weight	0.04	.84	−0.67	0.20
		Brood size * relative weight	11.21	<.01	0.14	0.04
3	IL‐6	Male sex	1.30	.25	0.01	0.01
		Brood size	0.98	.32	0.004	0.004
		Relative weight	1.69	.19	−0.04	0.17
		Brood size * relative weight	5.40	.02	0.008	0.003
4	hsCRP	Male sex	1.07	.30	0.10	0.10
		Brood size	0.18	.67	−0.03	0.06
		Relative weight	0.14	.71	0.18	0.30
		Brood size * relative weight	0.31	.58	−0.03	0.06

Where there were significant interactions between brood size and relative weights, we conducted simple slope analyses to interpret these interactions, using the methods presented by Preacher, Curran, and Bauer (2006). These methods allow the following: (1) visualization of the estimated slope of the relationship between the outcome variable and relative weight at every value of brood size; and (2) the identification of “regions of significance.” These are ranges of brood size within which the slope of the relationship between the outcome variable and relative weight is significantly negative or significantly positive.

### 2.6 Ethical information

Fieldwork was carried with the permission of the landowners and with permits from Natural England (license number 2015/SCI/0006) and the UK Home Office (PPL 70/8089). No nests were left completely empty at any time, and nestling time out of nest was minimized. Measurements and sampling took place in a heated car. Nine nestlings (5.5%) died between day 4 and day 16, and a further eight (4.9%) did not fledge successfully after completion of the study. These loss rates are low compared to observational records of starling nests (Feare, 1984), suggesting that our interventions did not have a substantial negative impact on mortality.

## 3 Results

### 3.1 Description of sample

We obtained full series of weights for 155 nestlings from 34 nests, with a day 16 brood size range of 3 to 6 (5 broods of 3, 9 broods of 4, 16 broods of 5, 4 broods of 6). This was from a day 4 sample size of 164 nestlings. The nine nestlings that died had come one each from nine different nests (2 broods of 4, 2 broods of 5, and 5 broods of 6). Thus, mortality was elevated in the largest broods: of an initial 9 broods of 6, 5 broods reduced to 5 nestlings by day 16.

Of these, we successfully obtained telomere measurements at both day 4 and day 16 points from 115 nestlings from 33 nests. The loss of 40 nestlings from the telomere sample was due to inability to obtain a sufficient blood sample on day 4, or assay failure at one or both time points (see 2). For, hsCRP, we successfully obtained measures for 151 individuals (34 nests), but plasma volumes were insufficient to obtain an IL‐6 value in a number of cases (see 2), resulting in a sample size of 112 nestlings (34 nests) for IL‐6. In what follows we use the maximal possible sample for each analysis; hence, sample sizes vary from model to model. Assay failure was independent of day 16 body weight and brood size for all three outcome measures (binomial regressions with measure missingness as the outcome variable and day 16 weight and day 16 brood size as the predictors: telomere length change (D): body weight, LRT = 0.38, *p* = .54, brood size, LRT = 1.13, *p* = .29 IL‐6: body weight, LRT = 0.55, *p* = .46, brood size, LRT = 1.40, *p* = .24; hsCRP: body weight, LRT = 0.77, *p* = .38, brood size, LRT = 0.84, *p* = .36; IL‐6).

### 3.2 Nestling weights

**Figure 1:**
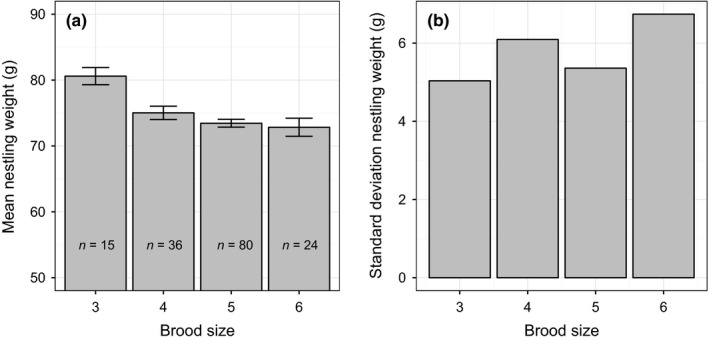
(a) Mean nestling weight at day 16 by brood size. Error bars represent one standard error. (b) Standard deviation of day 16 weight by brood size

Mean absolute nestling weights (g, standard deviation in parenthesis) were as follows: day 4, 19.51 (5.19); day 7, 45.17 (7.07), day 16 74.41 (6.08). We fitted a linear mixed model with day 16 weight as the outcome variable and brood size and sex as the fixed predictors (Table 1, model 1). Both effects were significant, with males heavier than females, and mean weight decreasing with increasing brood size (Table 1 and Figure 1a). Thus, our assumption that intrabrood competition for resources would be greater in the larger broods was supported. Marginal *r* ^2^ for this model was .17, with 42% of the remaining variation explained by nest (σ^2^_nest_ = 13.17, σ^2^_residual_ = 18.50). The variation in day 16 weight was smallest in the broods of three, and greatest in the broods of six, although variation did not increase consistently as brood size became larger (Figure 1b).

### 3.3 Telomere dynamics

**Figure 2:**
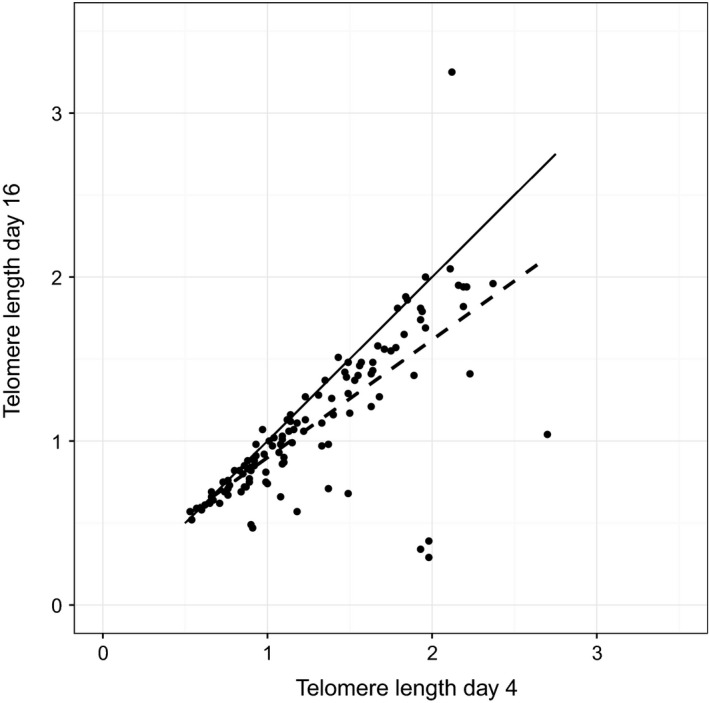
Relationship between telomere length on day 16 and telomere length on day 4 (T/S ratios). The dashed line represents the best fit through the data, whereas the solid line represents the y = x line, the line around which the data would be expected to fall if there were no change between day 4 and day 16

Telomere length on day 16 was highly correlated with telomere length on day 4 (*r*_113_ = .74, *p *<* *.01). Mean telomere lengths were 1.27 (0.49) on day 4 and 1.11 (0.47) on day 16. The shortening from day 4 to day 16 was significant (paired *t*‐test: t_114_ = −5.60, *p *<* *.01; 95/115 individuals had a lower value at day 16 than day 4). Figure 2 shows both the continuity, in the form of the positive association between day 4 and day 16 values, and the shortening, in that most observations fall below the y = x line.

To test for effects of body size and brood size on telomere loss, we fitted a model with telomere length change between the two time points (D) as the outcome. The key predictors were brood size, relative weight on day 16, and their interaction. As covariates, we included sex and, because telomere attrition has been previously observed to be faster in individuals with longer telomeres even after controlling for regression to the mean (Nettle et al., 2015; Verhulst et al., 2013), telomere length on day 4. Results were unchanged whether or not these covariates (whose effects were not significant) were included in the model. None of the main effects was significant (Table 1, model 2). However, there was a significant brood size by relative weight interaction. Marginal *r* ^2^ for this model was .07; 28.5% of the remaining variation was explained by nest, and none by assay plate (σ^2^_nest_ = 0.04, σ^2^_plate_ = 0.00; σ^2^_residual_ = 0.11).

**Figure 3:**
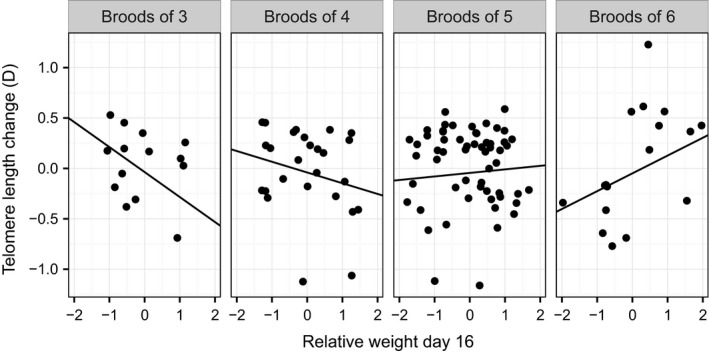
Telomere length change against relative weight at day 16 for each observed brood size. Lines represent estimates from the simple slopes analysis based on the full statistical model including random effects of nest and plate, and fixed effects of day 4 telomere length and sex. Points represent raw data points

To investigate the interaction between brood size and relative weight, we performed a simple slopes analysis. The slope of telomere change on relative weight was negative at a brood size of 3, and gradually increased until positive in brood sizes of 6 (Figure 3; simple slopes (and standard errors): brood size 3: −0.25 (0.08); brood size 4: −0.11 (0.05); brood size 5: 0.04 (0.04); brood size 6: 0.18 (0.06)). The region of significance analysis found the slopes to be significantly negative at brood sizes of 4.11 and smaller, and significantly positive at brood sizes of 5.34 and larger.

To establish that relative weight rather than absolute weight was indeed the better predictor of telomere length change, we compared model fits between the model described above, and a model in which relative day 16 weight was replaced with absolute day 16 weight. The results of the two models were qualitatively similar, but the model fit was better for the relative weight model (AIC 119.91) than the absolute weight model (AIC 122.46).

### 3.4 Immune markers

The correlation between the two immune markers was positive but marginally nonsignificant (*r*_119_ = .17, *p* = .07). The correlation between IL‐6 and telomere length change was also marginally nonsignificant, in the direction of higher IL‐6 activity, less telomere attrition (*r*_81_ = .21, *p* = .06). hsCRP was not significantly correlated with telomere length change (*r*_109_ = .13, *p* = .19).

With IL‐6 as the outcome variable, we fitted a model with brood size, relative weight on day 16, and the brood size by relative weight interaction as the key predictors (Table 1, model 3). Neither main effect was significant. However, the interaction was significant, again with a positive sign. Marginal *r* ^2^ for this model was .06; nest explained 31.0% of the remaining variation (σ^2^_nest_ = 0.00035, σ^2^_residual_ = 0.00079).

**Figure 4:**
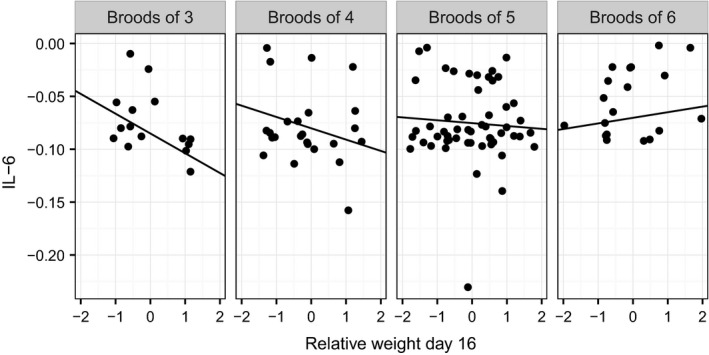
Plasma IL‐6 (reciprocally transformed and multiplied by −1) against relative weight at day 16 for each observed brood size. Lines represent estimates from the simple slopes analysis based on the full statistical model including a random effect of nest and a fixed effect of sex. Points represent raw data points

To interpret the interaction between brood size and relative weight, we again performed a simple slopes analysis. The estimated slopes again were from negative in the smallest broods and became more positive as brood size increased (Figure 4; simple slopes (standard errors): brood size 3, −0.02 (0.007); brood size 4, −0.01 (0.004); brood size 5, −0.003 (0.003); brood size 6, 0.005 (0.005)). The region of significance analysis found that the association between IL‐6 and relative weight was significantly negative for brood sizes of 4.55 and smaller and would become significantly positive if brood size was 8.47 or larger.

We repeated the model for IL‐6, but in place of relative weight using absolute weight. This produced similar results, but with a poorer model fit (AICs: relative weight: −438.48; absolute weight: −433.97).

For hsCRP, we again fitted a model with brood size, relative weight day 16, and the brood size by relative weight interaction as the key predictors (Table 1, model 4). Neither main effect nor the interaction was significant (marginal *r* ^2^ = .01). Nest explained 24.5% of the variation in hsCRP (σ^2^_nest_ = 0.17, σ^2^_residual_ = 0.53). In the absence of any significant interaction, simple slopes analysis was not pursued. The lack of significant predictors remained the case when absolute weight was used instead of relative weight.

## 4 Discussion

In this observational study of over 30 wild starling nests, we found that brood size moderated the association between relative size on day 16 and erythrocyte telomere length change. In small broods (four nestlings and smaller), the association was negative; that is, relatively larger birds showed more telomere loss. In large broods (six nestlings), the association was positive; the nestlings that were largest in their brood also showed the least telomere loss. Note that there was no main effect of brood size: Nestlings from larger broods did not show more telomere loss overall. Rather, only the combination of large brood size and small relative body size led to accelerated loss. For the immune marker IL‐6, we found a similar pattern. In broods of 3 or 4, the relatively smaller nestlings had higher IL‐6 levels. In larger broods, the association tended to become more positive, although our analysis suggests that it would only become significantly positive if brood size was to increase to over 8, which is beyond the natural range for starlings. Our other immune marker, hsCRP, showed no systematic patterning with brood size or growth.

The results were thus partially supportive of our main hypothesis. We predicted an interaction between brood size and relative nestling size in explaining the outcome variables, and the predicted interaction was indeed present for telomere length change and IL‐6. Thus, our results help reconcile previous experimental findings from jackdaws (Boonekamp et al., 2014) and starlings (Nettle et al., 2013) that the larger nestlings in large broods fare better than the smaller ones in terms of telomere attrition, with the negative associations between body size and telomere length observed in other field studies (Herborn et al., 2014; Noguera et al., 2015; Ringsby et al., 2015). These negative associations, in our data, are restricted to small broods, while positive associations appear in the largest broods. Thus, whether the overall association between body size and telomere loss in a study is negative, null, or positive may depend on the distribution of brood sizes in the sample.

Our assumption that brood size would be a reasonable marker of the intensity of within‐brood competition appeared justified by the fact that average day 16 weight declined with increase in brood size. This supports previous observations in the starling (Nettle et al., 2013; Wright & Cuthill, 1990b) and suggests that parents do not match their clutch sizes perfectly to their provisioning abilities; rather, on average, parents having larger broods expose their nestlings to reduced per capita food supply and greater competition than parents having smaller broods.

Our telomere data showed very similar patterns to our own previous studies in starlings (Nettle et al., 2013, 2015), and those observed in nestlings of other passerines (Boonekamp et al., 2014; Reichert et al., 2014; Stier et al., 2015). Erythrocyte telomere lengths at the two time points were strongly correlated with one another, indicating repeatable individual differences (this study, *r* = .74; Nettle et al., 2013; *r* = .72; Nettle et al., 2015; *r* = .90). However, the large majority of individuals had a lower value at the second time point, indicating an overall pattern of telomere shortening. The ability to detect significant within‐individual change over a period of just 12 days conforms to the pattern of rapid telomere shortening in early life that has been observed elsewhere (e.g., Heidinger et al., 2012).

The present study was purely observational, whereas much of the previous evidence concerning effects of nestling conditions on telomere or immune dynamics in birds has been based on experimental manipulation of brood size, position in the size hierarchy, or food supply (Birkhead et al., 1999; Boonekamp et al., 2014; Killpack et al., 2015; Nettle et al., 2013, 2015; Reichert et al., 2014). We see the two approaches as complementary. Taking the observational approach alone limits the inferences about causation that can be made. For example, the positive correlations between growth and immune function in the six broods could be due to variation among nestlings in genetic quality rather than the impact of competition. However, we can set the patterns observed here in the context of the evidence from the experimental studies, which clearly shows that factors such as relative size have a causal, not just correlative, significance for individual development (Nettle et al., 2015; Reichert et al., 2014). Experimental approaches alone are also incomplete, as it is necessary to show that the causal factors they expose are actually operative and significant in the range of natural, un‐manipulated broods.

Our immune measures suffered a number of limitations. There are a large number of different immune parameters that can be measured. Research in ecological immunology tends to be opportunistic in which it uses, based on which markers are available and feasible for nonmodel animals under field conditions (Demas & Carlton, 2015). This is true of our study. Nonetheless, cytokines such as IL‐6 in particular provide relatively direct and precise measures of immune activity (Zimmerman et al., 2014), and there is some evidence that in nestlings, different immune measures tend to be correlated with one another (Hoi‐Leitner et al., 2001), suggesting that our conclusions might have been similar had we chosen different assays. However, our hsCRP assay was extremely weakly correlated with IL‐6, despite IL‐6 stimulating the production of C‐reactive protein in the adult immune system. Another limitation of our immune measures was that we measured baseline levels rather than the response to an experimental challenge, as is more usually performed in studies of immune capacity. We would nonetheless defend our assay as meaningful. All bird nests are highly pathogenic environments (Brandl et al., 2014; Goodenough & Stallwood, 2012). Immune capacity is initially low in hatchlings and increases rapidly, but does not reach adult levels by fledging (Killpack & Karasov, 2012; Stambaugh et al., 2011). Thus, it is a reasonable assumption that variation across nestlings in the extent of cytokine activity primarily reflects variation in the development of immune capacity, especially when comparing individuals from the same nest.

Although trade‐offs between different components of development are a central component of life‐history theory, the negative correlations they imply are often difficult to detect phenotypically. Generally, this is due to inequality between individuals in available resources, which masks any underlying trade‐offs (van Noordwijk & De Jong, 1986). This study shows how the competitive context—specifically the number of other nest‐mates—is crucial in determining the extent to which being larger simply allows young birds to fare better on all fronts.

## Conflict of Interest

None declared.

## Data Accessibility

Raw data and an R script for analysis are uploaded with this manuscript as Supporting information.

## References

[ece32551-bib-0001] Abd El‐Hamid, H. S. , Ahmed, H. A. , Sadek, K. M. , & Ellakany, H. F. (2014). Impact of serratiopeptidase treatment on performance and health parameters in broiler chickens. International Journal of Pharmaceutical Sciences Review and Research, 26, 84–90.

[ece32551-bib-0002] Adelman, J. S. , Bentley, G. E. , Wingfield, J. C. , Martin, L. B. , & Hau, M. (2010). Population differences in fever and sickness behaviors in a wild passerine: A role for cytokines. The Journal of Experimental Biology, 213, 4099–4109. 21075952 10.1242/jeb.049528

[ece32551-bib-0003] Bartke, A. (2012). Healthy aging: Is smaller better? ‐ A mini‐review. Gerontology, 58, 337–343. 22261798 10.1159/000335166 PMC3893695

[ece32551-bib-0004] Bates, D. , Machler, M. , Bolker, B. , & Walker, S. (2015). Fitting linear mixed‐effects models using lme4. Journal of Statistical Software, 67, 1–48.

[ece32551-bib-0005] Bernstein, R. M. (2010). The big and small of it: How body size evolves. American Journal of Physical Anthropology, 143, 46–62. 21086526 10.1002/ajpa.21440

[ece32551-bib-0006] Birkhead, T. R. , Fletcher, F. , & Pellatt, E. J. (1999). Nestling diet, secondary sexual traits and fitness in the zebra finch. Proceedings of the Royal Society B: Biological Sciences, 266, 385.

[ece32551-bib-0007] Bize, P. , Criscuolo, F. , Metcalfe, N. B. , Nasir, L. , & Monaghan, P. (2009). Telomere dynamics rather than age predict life expectancy in the wild. Proceedings of the Royal Society B: Biological Sciences, 276, 1679–1683. 19324831 10.1098/rspb.2008.1817 PMC2660992

[ece32551-bib-0008] Boonekamp, J. J. , Mulder, G. A. , Salomons, H. M. , Dijkstra, C. , & Verhulst, S. (2014). Nestling telomere shortening, but not telomere length, reflects developmental stress and predicts survival in wild birds. Proceedings of the Royal Society B‐Biological Sciences B: Biological Sciences, 281, 20133287. 10.1098/rspb.2013.3287 PMC4024283 24789893

[ece32551-bib-0009] Brandl, H. B. , Van Dongen, W. F. D. , Darolova, A. , Kristofik, J. , Majtan, J. , & Hoi, H. (2014). Composition of bacterial assemblages in different components of reed warbler nests and a possible role of egg incubation in pathogen regulation. PLoS One, 9, 1–19. 10.1371/journal.pone.0114861 PMC4262450 25493434

[ece32551-bib-0010] Brommer, J. E. (2004). Immunocompetence and its costs during development: An experimental study in blue tit nestlings. Proceedings of the Royal Society of London B: Biological Sciences, 271(Suppl), S110–S113. 10.1098/rsbl.2003.0103 PMC1809982 15101435

[ece32551-bib-0011] Cawthon, R. M. (2002). Telomere measurement by quantitative PCR. Nucleic Acids Research, 30, 1–6. 12000852 10.1093/nar/30.10.e47 PMC115301

[ece32551-bib-0012] Cotton, P. A. , Wright, J. , & Kacelnik, A. (1999). Chick begging strategies in relation to brood hierarchies and hatching asynchrony. American Naturalist, 153, 412–420. 10.1086/303178 29586619

[ece32551-bib-0013] Criscuolo, F. , Bize, P. , Nasir, L. , Metcalfe, N. B. , Foote, C. G. , Griffiths, K. , … Monaghan, P. (2009). Real‐time quantitative PCR assay for measurement of avian telomeres. Journal of Avian Biology, 40, 342–347.

[ece32551-bib-0014] Demas, G. E. , & Carlton, E. D. (2015). Ecoimmunology for psychoneuroimmunologists: Considering context in neuroendocrine‐immune‐behavior interactions. Brain, Behavior, and Immunity, 44C, 9–16. 10.1016/j.bbi.2014.09.002 PMC4275338 25218837

[ece32551-bib-0015] Fair, J. M. , Hansen, E. S. , & Ricklefs, R. E. (1999). Growth, developmental stability, and immune response in juvenile Japanese quail (Coturnix coturnix japonica). Proceedings of the Royal Society of London B: Biological Sciences, 266, 1735–1742. 10.1098/rspb.1999.0840 PMC1690192 10518322

[ece32551-bib-0016] Feare, C. (1984). The starling. Oxford, UK: Oxford University Press.

[ece32551-bib-0017] Fridsolfsson, A.‐K. , & Ellegren, H. (1999). A simple and universal method for molecular sexing of non‐ratite birds. Journal of Avian Biology, 30, 116–121.

[ece32551-bib-0018] Goodenough, A. E. , & Stallwood, B. (2012). Differences in culturable microbial communities in bird nestboxes according to orientation and influences on offspring quality in great tits (Parus major). Microbial Ecology, 63, 986–995. 22183046 10.1007/s00248-011-9992-7

[ece32551-bib-0019] Hasselquist, D. , & Nilsson, J. Å. (2012). Physiological mechanisms mediating costs of immune responses: What can we learn from studies of birds? Animal Behaviour, 83, 1303–1312.

[ece32551-bib-0020] Heidinger, B. J. , Blount, J. D. , Boner, W. , Griffiths, K. , Metcalfe, N. B. , & Monaghan, P. (2012). Telomere length in early life predicts lifespan. Proceedings of the National Academy of Sciences of the United States of America, 109, 1743–1748. 22232671 10.1073/pnas.1113306109 PMC3277142

[ece32551-bib-0021] Herborn, K. A. , Heidinger, B. J. , Boner, W. , Noguera, J. C. , Adam, A. , Daunt, F. , & Monaghan, P. (2014). Stress exposure in early post‐natal life reduces telomere length : An experimental demonstration in a long‐lived seabird. Proceedings Of The Royal Society B‐Biological Sciences, 281, 20133151. 10.1098/rspb.2013.3151 PMC3973262 24648221

[ece32551-bib-0022] Hoeijmakers, J. H. J. (2001). Genome maintenance mechanisms for preventing cancer. Nature, 411, 366–374. 11357144 10.1038/35077232

[ece32551-bib-0023] Hoi‐Leitner, M. , Romero‐Pujante, M. , Hoi, H. , & Pavlova, A. (2001). Food availability and immune capacity in serin (Serinus serinus) nestlings. Behavioral Ecology and Sociobiology, 49, 333–339.

[ece32551-bib-0024] Killpack, T. L. , Elijah, C. , & Karasov, W. H. (2015). Impacts of short‐term food restriction on immune development in altricial house sparrow nestlings. Physiological and Biochemical Zoology, 88, 195–207. 25730274 10.1086/680168

[ece32551-bib-0025] Killpack, T. L. , & Karasov, W. H. (2012). Ontogeny of adaptive antibody response to a model antigen in captive altricial zebra finches. PLoS One, 7, e47294. 23056621 10.1371/journal.pone.0047294 PMC3467253

[ece32551-bib-0026] Kilner, R. (1995). When do canary parents respond to nestling signals of need? Proceedings of the Royal Society B: Biological Sciences, 260, 343–348.

[ece32551-bib-0027] Kilner, R. (2001). A growth cost of begging in captive canary chicks. Proceedings of the National Academy of Sciences of the United States of America, 98, 11394–11398. 11572988 10.1073/pnas.191221798 PMC58740

[ece32551-bib-0028] Lochmiller, R. L. , Vestey, M. R. , & Boren, J. C. (1993). Relationship between protein nutritional‐status and immunocompetence in Northern bobwhite chicks. Auk, 110, 503–510.

[ece32551-bib-0029] Miller, R. A. , Harper, J. M. , Galecki, A. , & Burke, D. T. (2002). Big mice die young: Early life body weight predicts longevity in genetically heterogeneous mice. Aging Cell, 1, 22–29. 12882350 10.1046/j.1474-9728.2002.00006.x

[ece32551-bib-0030] Mock, D. W. , Schwagmeyer, P. L. , & Dugas, M. B. (2009). Parental provisioning and nestling mortality in house sparrows. Animal Behaviour, 78, 677–684.

[ece32551-bib-0031] Nakagawa, S. , & Schielzeth, H. (2013). A general and simple method for obtaining R^2^ from generalized linear mixed‐effects models. Methods in Ecology and Evolution, 4, 133–142.

[ece32551-bib-0032] Nettle, D. (2014). What the future held: Childhood psychosocial adversity is associated with health deterioration through adulthood in a cohort of British women. Evolution and Human Behavior, 35, 519–525.

[ece32551-bib-0033] Nettle, D. , Monaghan, P. , Boner, W. , Gillespie, R. , & Bateson, M. (2013). Bottom of the heap: Having heavier competitors accelerates early‐life telomere loss in the european starling, Sturnus vulgaris. PLoS One, 8, e83617. 24386235 10.1371/journal.pone.0083617 PMC3873947

[ece32551-bib-0034] Nettle, D. , Monaghan, P. , Gillespie, R. , Brilot, B. , Bedford, T. , & Bateson, M. (2015). An experimental demonstration that early‐life competitive disadvantage accelerates telomere loss. Proceedings of the Royal Society B: Biological Sciences, 282, 20141610. 25411450 10.1098/rspb.2014.1610 PMC4262165

[ece32551-bib-0035] Noguera, J. , Metcalfe, N. , Boner, W. , & Monaghan, P. (2015). Sex‐dependent effects of nutrition on telomere dynamics in zebra finches (Taeniopygia guttata). Biology Letters, 11, 20140938. 25716087 10.1098/rsbl.2014.0938 PMC4360105

[ece32551-bib-0036] van Noordwijk, A. J. , & De Jong, G. (1986). Acquisition and allocation of resources: Their influence on variation in life history tactics. American Naturalist, 128, 137–142.

[ece32551-bib-0037] Nussey, D. H. , Baird, D. , Barrett, E. , Boner, W. , Fairlie, J. , Gemmell, N. , … Monaghan, P. (2014). Measuring telomere length and telomere dynamics in evolutionary biology and ecology. Methods in Ecology and Evolution, 5, 299–310. 25834722 10.1111/2041-210X.12161 PMC4375921

[ece32551-bib-0038] Pepys, M. B. , & Hirschfield, G. M. (2003). C‐reactive protein: A critical update. Journal of Clinical Investigation, 111, 1805–1812. 12813013 10.1172/JCI18921 PMC161431

[ece32551-bib-0039] Pfaffl, M. W. (2001). A new mathematical model for relative quantification in real‐time RT‐PCR. Nucleic Acids Research, 29, 16–21. 10.1093/nar/29.9.e45 PMC55695 11328886

[ece32551-bib-0040] Preacher, K. J. , Curran, P. J. , & Bauer, D. J. (2006). Computational tools for probing interactions in multiple linear regression, multilevel modeling, and latent curve analysis. Journal of Educational and Behavioral Statistics, 31, 437–448.

[ece32551-bib-0041] Quirici, V. , Guerrero, C. J. , Krause, J. S. , Wingfield, J. C. , & Vásquez, R. A. (2016). The relationship of telomere length to baseline corticosterone levels in nestlings of an altricial passerine bird in natural populations. Frontiers in Zoology, 13, 1–11. 26759601 10.1186/s12983-016-0133-5 PMC4710010

[ece32551-bib-0042] R Core Development Team . (2015) R: A Language and Environment for Statistical Computing.

[ece32551-bib-0043] Reichert, S. , Criscuolo, F. , Zahn, S. , Arrive, M. , Bize, P. , & Massemin, S. (2014). Immediate and delayed effects of growth conditions on ageing parameters in nestling zebra finches. Journal of Experimental Biology, 218, 491–499. 25524985 10.1242/jeb.109942

[ece32551-bib-0044] Rhymer, C. M. , Devereux, C. L. , Denny, M. J. H. , & Whittingham, M. J. (2012). Diet of Starling Sturnus vulgaris nestlings on farmland: The importance of Tipulidae larvae. Bird Study, 59, 426–436.

[ece32551-bib-0045] Ringsby, T. H. , Jensen, H. , Pärn, H. , Kvalnes, T. , Boner, W. , Gillespie, R. , … Monaghan, P. (2015). On being the right size: Increased body size is associated with reduced telomere length under natural conditions. Proceedings of the Royal Society B: Biological Sciences, 282, 20152331. 26631569 10.1098/rspb.2015.2331 PMC4685786

[ece32551-bib-0046] Saino, N. , Calza, S. , & Moller, A. P. (1997). Immunocompetence of nestling barn swallows in relation to brood size and parental effort. Journal of Animal Ecology, 66, 827–836.

[ece32551-bib-0047] Sheldon, B. C. , & Verhulst, S. (1996). Ecological immunology: Costly parasite defences and trade‐offs in evolutionary ecology. Trends in Ecology and Evolution, 11, 317–321. 21237861 10.1016/0169-5347(96)10039-2

[ece32551-bib-0048] Snoeijs, T. , Pinxten, R. , & Eens, M. (2005). Experimental removal of the male parent negatively affects growth and immunocompetence in nestling great tits. Oecologia, 145, 165–173. 15891821 10.1007/s00442-005-0088-2

[ece32551-bib-0049] Stambaugh, T. , Houdek, B. J. , Lombardo, M. P. , Thorpe, P. A. , & Caldwell Hahn, D. (2011). Innate immune response development in nestling tree swallows. The Wilson Journal of Ornithology, 123, 779–787.

[ece32551-bib-0050] Stier, A. , Massemin, S. , Zahn, S. , Tissier, M. L. , & Criscuolo, F. (2015). Starting with a handicap: Effects of asynchronous hatching on growth rate, oxidative stress and telomere dynamics in free‐living great tits. Oecologia, 179, 999–1010. 26314343 10.1007/s00442-015-3429-9

[ece32551-bib-0051] Tella, J. L. , Bortolotti, G. R. , Forero, M. G. , & Dawson, R. (2000). Environmental and genetic variation in T‐cell‐mediated immune response of fledging American kestrels. Oecologia, 123, 453–459. 10.1007/s004420000331 28308752

[ece32551-bib-0052] Van Der Most, P. J. , De Jong, B. , Parmentier, H. K. , & Verhulst, S. (2011). Trade‐off between growth and immune function: A meta‐analysis of selection experiments. Functional Ecology, 25, 74–80.

[ece32551-bib-0053] Verhulst, S. , Aviv, A. , Benetos, A. , Berenson, G. S. , & Kark, J. D. (2013). Do leukocyte telomere length dynamics depend on baseline telomere length? An analysis that corrects for “regression to the mean”. European Journal of Epidemiology, 28, 859–866. 23990212 10.1007/s10654-013-9845-4

[ece32551-bib-0054] Von Zglinicki, T. (2002). Oxidative stress shortens telomeres. Trends in Biochemical Sciences, 27, 339–344. 12114022 10.1016/s0968-0004(02)02110-2

[ece32551-bib-0055] Wright, J. , & Cuthill, I. (1990a). Biparental care: Short‐term manipulation of partner contribution and brood size in the starling, Sturnus vulgaris. Behavioral Ecology, 1, 116–124.

[ece32551-bib-0056] Wright, J. , & Cuthill, I. (1990b). Manipulation of sex differences in parental care: The effect of brood size. Animal Behaviour, 40, 462–471.

[ece32551-bib-0057] Zimmerman, L. M. , Bowden, R. M. , & Vogel, L. A. (2014). A vertebrate cytokine primer for eco‐immunologists. Functional Ecology, 28, 1061–1073.

